# Hospitals and Dental Service

**Published:** 1919-06-28

**Authors:** 


### HOSPITALS AND DENTAL SERVICE.

The exact relation of faulty and carious teeth to systemic disease is still a matter upon which occurs considerable disagreement. On the one hand, we have those who point to the number of people who pass relatively healthily through life, or are actually cured of disease, having meanwhile a thoroughly unsatisfactory dentition. On the other, we have the enthusiasts who claim pyorrhœa and caries as the root cause of the majority of sickness which besets mankind.

Here, however, it is not our object to contrast the value of these views, for there are many well-known truths regarding the "dangers of oral sepsis" which none will deny. Rather would we point out that in many of our general hospitals, even where elaborate dental establishments are found, relatively poor attention is given to the dental care of hundreds of patients, although full realisation exists that one of the most important problems in improving our C 3 population lies in general efficiency of care for the nation's teeth. It is perhaps another instance of the need for full-time hospital staffs, for taking the instance of a certain American hospital, some records of which come to hand, we read of an expert dental clinician in full time attendance and of in- and out-patients, each with a hospital card upon which his tooth condition and treatment are minutely detailed by this hospital officer.

Apart from the fact that his findings frequently furnish invaluable data to the doctor, there is another far more important service. It is the ideal function of a hospital not only to place a patient on his feet but to restore him if possible to his normal self and full usefulness, and even if the dental deficiencies cannot be said in any way to account for the immediate illness, yet, while under institutional control, it is an invaluable asset to the general health of the public if each patient can be discharged with both dental treatment and the need of it brought actively to his notice.

Particularly does this apply to children. But, to take another general consideration, whatever view of oral sepsis in relation to disease we may now take, there is no doubt that an enormous amount of research work in pathology and bacteriology of mouth conditions remains to be done, and that true co-ordination of medical and dental investigations can be obtained only by some such in-patient methods. One knows that it is an everyday procedure to hand on a hospital case for treatment in the dental department, but too often the only connecting link is a note, "Will dental surgeon kindly treat?" and no true interchange of ideas or results occurs between the departments. If no thought of such dental need occurs to the physician or surgeon the patient may never have even the scantiest examination of his mouth in spite of the fact that every possible accessory for dental care is immediately at hand. Annually thousands of people with mouths indescribably septic leave our hospitals without treatment or any arrangements for obtaining it.

One may say confidently that clearing up of mouth conditions almost invariably accelerates the recovery from systemic disease, and, we believe, this is generally realised and accepted. We speak nowadays so frequently of prevention. Would not routine care of at least all in-patients' teeth be one small step towards its attainment in many branches of both medical and surgical disease?

